# Phytotoxicity, Bioaccumulation, and Degradation of Nonylphenol in Different Microalgal Species without Bacterial Influences

**DOI:** 10.3390/ijms21041338

**Published:** 2020-02-17

**Authors:** Ning He, Zhiwei Liu, Xian Sun, Shuangyao Wang, Weijie Liu, Dong Sun, Shunshan Duan

**Affiliations:** 1 College of Life Science and Resources and Environment, Yichun University, Yichun 336000, China; hening2010@163.com; 2 School of Environment and Energy, South China University of Technology, Guangzhou 510006, China; zwliumost@126.com; 3 Southern Marine Science and Engineering Guangdong Laboratory (Zhuhai), Zhuhai Key Laboratory of Marine Bioresources and Environment, Guangdong Provincial Key Laboratory of Marine Resources and Coastal Engineering, School of Marine Sciences, Sun Yat-Sen University, Guangzhou 510275, China; 4 Institute for Marine & Antarctic Studies, University of Tasmania, Private Bag 49, Hobart, TAS 7001, Australia; shuangyaowang1314@outlook.com; 5 South China Institute of Environmental Science, Ministry of Ecology and Environment, NO.18 Ruihe RD., Guangzhou 510535, China; liuweijie@scies.org; 6 Institute of Hydrobiology, Jinan University, Guangzhou 510632, China; jnu_sundong@163.com (D.S.); tssduan@jnu.edu.cn (S.D.)

**Keywords:** nonylphenol, microalgae, photosynthetic activities, antioxidant enzyme, biodegradation

## Abstract

Nonylphenol (NP) is a contaminant that has negative impacts on aquatic organisms. To investigate its phytotoxicity, bioaccumulation, and degradation in algae without associated bacteria, six freshwater microalgae—*Ankistrodesmus acicularis, Chlorella vulgaris*, *Chroococcus minutus*, *Scenedesmus obliquus*, *Scenedesmus quadricauda*, and *Selenastrum bibraianum*—in bacteria-free cultures were studied. When exposed to 0.5–3.0 mg L^−1^ NP for 4 days, cell growth and photosynthesis, including maximal photochemistry (*Fv/Fm*), were suppressed progressively. The antioxidant responses of superoxide dismutase (SOD), catalase (CAT), and peroxidase (POD) showed species differences. While the antioxidant enzymes in *C. vulgaris* and *S. obliquus* were more active with the increase of NP (0–3 mg L^−1^), they dropped in the other four algae at concentrations of 1 and 1.5 mg L^−1^. Therefore, *C. vulgaris* and *S. obliquus* were designated as NP-tolerant species and showed more conspicuous and faster changes of antioxidant reactions compared with the four NP-sensitive species. All six species degraded NP, but *A. acicularis* was more reactive at low NP concentrations (<1 mg L^−1^), suggesting its possible application in sewage treatment for its potential for effective NP removal from water bodies in a suitable scope. Therefore, the conclusion is that biodegradation of NP by algae is species specific.

## 1. Introduction

Nonylphenol (NP) is a common contaminant widely used in industrial, commercial, and household products such as detergents; emulsifiers; wetting, dispersing, and antistatic agents; demulsifiers; and solubilizers [1,2]. It is a microbial biodegradation product of nonylphenol ethoxylates (NPEOs) and is persistent, toxic, and disrupts endocrine function. NP has frequently been detected in rivers, lakes, ocean sediments, and soils. Although it is forbidden by the European Union to use NP or its ethoxylates [3], levels from 10 ng L^−1^ to over 100 ug L^−1^ have still been detected in the United States, China, and Japan, especially in polluted areas [4,5,6,7].

The mechanism by which NP is toxic to aquatic animals is not understood. Microalgae have simple life histories and maintain the balance of aquatic ecosystems. They have the potential to take up and degrade contaminants such as herbicides, pesticides, and phenols [8,9]. Our knowledge of the impact of NP on algae is lacking. Of special interest are those algae which are useful for biological research in nutrient enrichment, organic contamination, heavy metals, and various other stresses [10,11,12,13,14,15,16,17]. Therefore, we consider research on the toxicity of and adaption to NP biodegradation in unicellular algae worthwhile.

In nature, microalgae are associated with specific bacteria called the algal microbiome, which plays a critical role in modulating algal populations. Such a microbiome makes it difficult to assess the relationship between microalgae and NP, as the associated bacteria can degrade NP under aerobic conditions [18,19]. Hence, elimination of bacteria from stock algal cultures is important in order to study the toxicity and degradation of NP by microalgae.

Our previous study of removal and biodegradation of NP using microalgae (such as *Scenedesmus quadricauda*, *Ankistrodesmus acicularis*, *Chlorella vulgaris*, and *Chroococcus minutus*) focused on the growth, removal, and biodegradation rates [20]. Scant data, however, have showed the phytotoxicity effects of NP on various microalgae species. Therefore, a better understanding of physiological irritability variation in NP exposure of microalgae is needed. This study aimed to illustrate the phytotoxicity of NP and its accumulation in six freshwater microalgae obtained from NP-polluted water. We exposed five green algae (*Chlorella vulgaris* JNU38, *Scenedesmus obliquus* JNU15, *Selenastrum bibraianum* JNU28, *Ankistrodesmus acicularis* JNU14, and *Scenedesmus quadricauda* JNU39) and one cyanobacterium (*Chroococcus minutus* JNU17) from bacteria-free cultures to NP. The growth, fluorescence, antioxidant enzyme activity, accumulation of NP, degradation of adsorbed NP, as well as the ability to eliminate NP from the medium were determined. This work establishes the ecotoxicology of unicellular algae without bacterial influence under different NP levels and the adaptive response to organic xenobiotics.

## 2. Results and Discussion

### 2.1. Affect of NP on Algal Growth

**Figure 1:**
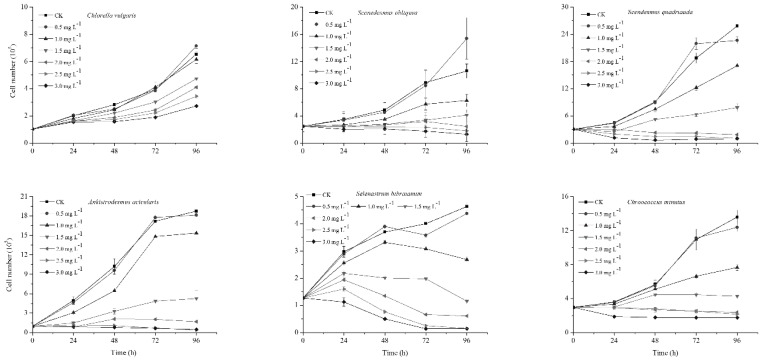
Effect of nonylphenol (NP) on the cell number of microalgae. Algae were treated with NP at 0–3.0 mg L^−1^ culture for 96 h. Values are the mean ± standard deviation (SD) (*n* = 3).

The effects of NP on the growth of *C. vulgaris*, *S. obliquus*, *S. bibraianum*, *A. acicularis*, *S. quadricauda*, and *C. minutus* under different NP concentrations (0.5, 1.0, 1.5, 2.0, 2.5, and 3.0 mg L^−1^) depended on species, exposure times, and concentrations (Figure 1). Although *S. bibraianum* and *C. minutus* showed different drops in growth at 96 h compared with the control, NP at levels between 0 and 0.5 mg L^−1^ had no influence on growth after 0–72 h exposure. The increase in cell density at low NP concentrations demonstrated the “poison exciting effect (hormesis)” [21]. However, the growth patterns among species varied at NP concentrations from 1.0 to 3.0 mg L^−1^. At 1.0 mg L^−1^, cell density in *C. vulgaris* exhibited a slight decrease at 96 h compared with the control (Figure 1). At 1.0-3.0 mg L^−1^, the cell densities of *S. obliquus*, *S. bibraianum*, *A. acicularis*, *S. quadricauda*, and *C. minutus* dropped at all exposure times, and there were negative correlations between cell density and NP levels. High concentrations of NP reduced algal growth and the toxicity of NP increased with exposure time (Figure 1). For *C. vulgaris*, *S. obliquus*, *S. bibraianum*, *A. acicularis*, *S. quadricauda*, and *C. minutus*, cell density at 3.0 mg L^−1^ of NP following 4 days of exposure reduced to 41.67%, 12.21%, 3.83%, 2.59%, 3.00%, and 12.85% of the controls, respectively. *C. vulgaris* showed the highest tolerance to NP, followed by *S. obliquus*, while the other algae were more sensitive to NP than *C. vulgaris* and *S. obliquus*.

Microalgae withstand damage by organic pollutants by many mechanisms. For example, cell walls containing a high amount of carbohydrates and proteins serve as barriers [22]. *C. vulgaris* produces extra cell wall polysaccharides to cope with high concentrations of pollutants [23]. Further, tolerance to oxidative stress plays a vital role as a defense mechanism [24,25].

A regression equation of NP concentration with the growth inhibition rate was obtained (Table 1). Logistic NP concentrations and inhibitory rate in the present study exhibited a dosage–response relationship. The respective 96 h median inhibitory effect concentration (EC_50_) values of NP to *C. vulgaris*, *S. obliquus*, *S. bibraianum*, *A. acicularis*, *S. quadricauda*, and *C. minutus* were 1.534, 1.179, 1.177, 1.100, 1.080, and 1.005 mg L^−1^ (Table 1). The EC_50_ values of NP to *C. vulgaris* were much higher than those of other algae. The lowest EC_50_ values of NP indicated its higher toxicity to *C. minutus*. Previous studies reported that the EC_50_ of NP varied from 0.017 to 3.00 mg L^−1^ to teleosts, and 0.021 to 3.00 mg L^−1^ to invertebrates [26]. A similar result showed that the 96 h EC_50_ of NP was 1.01–1.53 mg L^−1^. The 96 h EC_50_ of NP for *C. minutus* was 1.01 mg L^−1^, illustrating that *C. minutus* was more sensitive than the other species (Table 2).

### 2.2. Influence of NP on Chlorophyll Fluorescence

**Figure 2:**
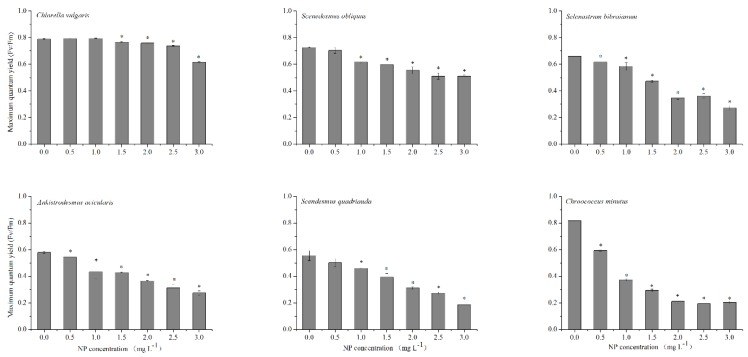
Effect of NP on the maximal PSII activity (*Fv/Fm*) in microalgae. Algae were treated with NP at 0–3.0 mg L^−1^ culture for 96 h. Mean and standard deviation of three replicates are shown. Values are the mean ± standard deviation (SD) (*n* = 3). Asterisks indicate the significant differences between the NP treatments and control (*p* < 0.05).

Maximal photochemistry (*Fv/Fm*), which originates mainly from the chlorophyll of PSII and illustrates the chlorophyll fluorescence emission of photosynthetic active organisms, has been broadly applied to elucidate environmental stresses [34]. When exposed to high levels of NP (1.5–3.0 mg L^−1^) after 96 h, *Fv/Fm* of the six algae were all significantly reduced, while *Fv/Fm* in *C. vulgaris* was noticeably higher compared with other algae (*p* < 0.05) (Figure 2). *Fv/Fm* of *S. obliquus*, *S. bibraianum*, *A. acicularis*, *S. quadricauda*, and *C. minutus* were significantly reduced with the increase of NP concentrations (*p* < 0.05). However, *Fv/Fm* in *C. vulgaris* at low NP concentrations (1.0–1.5 mg L^−1^) was insignificant (Figure 2). *Fv/Fm* in *C. vulgaris*, *S. obliquus*, *S. bibraianum*, *A. acicularis*, *S. quadricauda*, and *C. minutus* at 3.0 mg L^−1^ of NP were 77.9%, 70.3%, 41.2%, 47.4%, 33.6%, and 25.0% of their respective controls. Highly toxic pollutants damage the PSII system, resulting in a decrease of *Fv/Fm* and inducing a strong inhibition of photosynthetic electron transport, as demonstrated by the decrease of ΦsPSII [12]. Further, NP concentrations higher than 1.5 mg L^−1^ damaged algal cells, but NP below 1.5 mg L^−1^ had an undetectable influence on *C. vulgaris*. In summary, exposure of algae to NP levels higher than 1.5 mg L^−1^ inactivated PSII reaction centers and thus suppressed electron transport in PSII. By comparison, *C. vulgaris* exhibited higher tolerance to low NP levels. This corresponded to the estimation of EC_50_.

### 2.3. The Relationship between NP and Antioxidant Enzymes

As demonstrated by previous studies, oxidative stress may be partially responsible for the toxicity of NP due to the promotion of the generation of reactive oxygen species (ROS) and/or inhibition of the antioxidant system [35]. An excessive amount of ROS harms plants, including algae, by reacting to biomolecules at varying degrees and by direct damage to proteins, amino acids, nucleic acids, porphyrins, phenolic substances, and so forth [36]. To defend against the ROS-caused deleterious effects resulting from cellular oxidative stress, antioxidant enzymes play vital roles in the antioxidant system. Therefore, changes in antioxidants induced by NP reflect the toxicity of NP.

**Figure 3:**
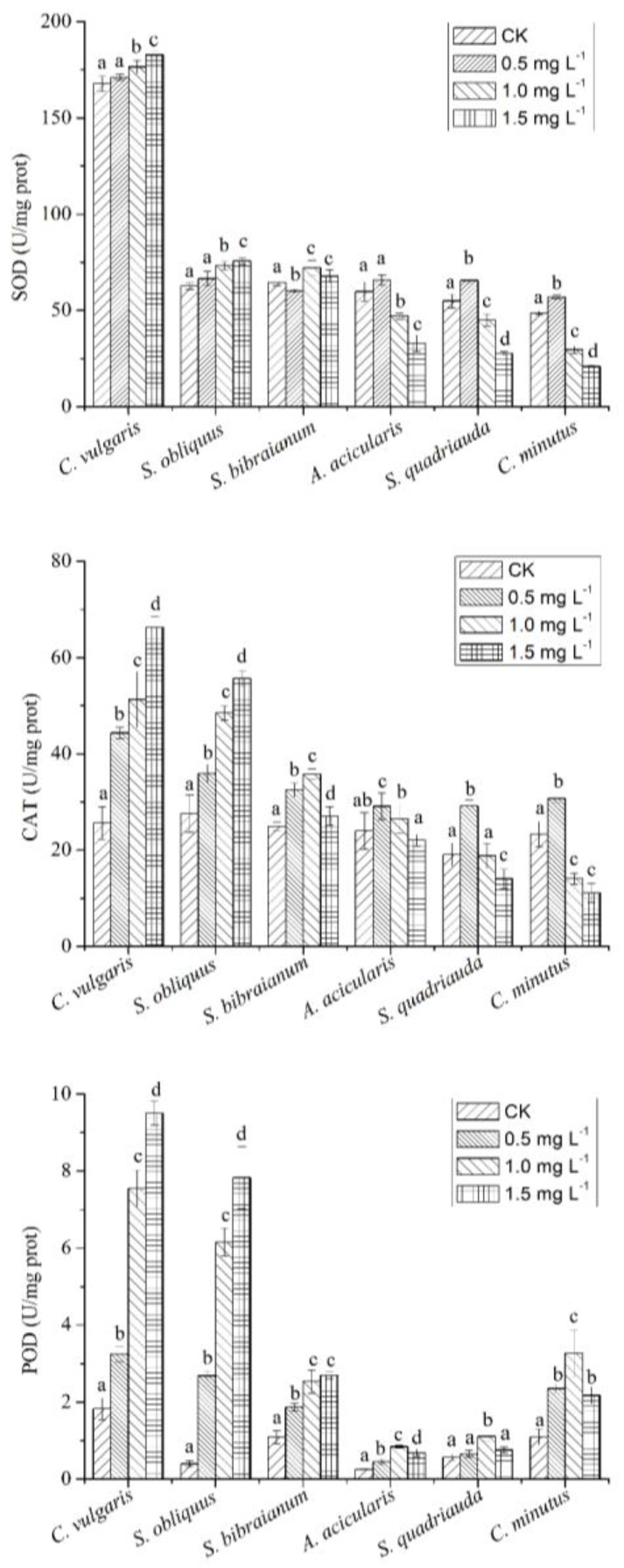
Effects of NP on activities of superoxide dismutase (SOD), catalase (CAT), and peroxidase (POD) in microalgae. Algae were treated with NP at 0.5, 1.0, and 1.5 mg L^−1^ culture for 96 h, and then the activities were assayed. Mean and standard deviation of three replicates are shown. Means with different letters at each NP concentration for each algal species indicate that they were significantly different at *p* < 0.05 according to a one-way ANOVA test. NS: not significant.

Algae respond to oxidative stress by strengthening the antioxidant defense systems, especially by inducing antioxidant enzymes [37]. Superoxide dismutase (SOD), the first line of defense, catalyzes dismutation of O^•^_2_ to H_2_O_2_. The drop of SOD depends on both NP concentration and algal species (Figure 3). SOD in *C. vulgaris* in the control group was significantly lower in comparison with other groups (*p* < 0.001). SOD in *C. vulgaris* was the highest, followed by *S. obliquus*, *S. bibraianum*, *A. acicularis*, *S. quadricauda*, and *C. minutus* (Figure 3), which was consistent with EC_50_ values. For *S. bibraianum*, *C. vulgaris*, and *S. obliques* exposed to 1.5 mg L^−1^ of NP, the activity of SOD increased by 5%, 9%, and 21%, respectively, compared with their control (*p* < 0.05) (Figure 3). For *A. acicularis*, *S. quadricauda*, and *C. minutus*, SOD exhibited slight increases with the rise of NP. However, when the NP level was high, SOD was reduced by 45%, 50%, and 57%, respectively, in comparison with their control (*p* < 0.05) (Figure 3), probably because the protein structure was damaged.

Hydrogen peroxide (H_2_O_2_) is a highly toxic by-product in SOD-mediated reactions. Its level, therefore, should be under tight control [38,39]. Catalase (CAT) is one of the key enzymes that scavenge H_2_O_2_. The highest CAT activity in this work was observed in *C. vulgaris*, followed by *S. obliquus*, *S. bibraianum*, *A. acicularis*, and *S. quadricauda*, and the lowest activity was presented in *C. minutus* (Figure 2), which was consistent with EC_50_.

The most significant change in CAT was found in *C. vulgaris* at 1.5 mg L^−1^ NP, with a 2.59-fold increase in comparison with the control treatment. CAT in *C. vulgaris* and *S. obliquus* showed an increase at all NP treatments. CAT in *A. acicularis*, *S. quadricauda*, and *C. minutus* presented a noticeable increase at low NP but was obviously suppressed at 1.0–1.5 mg L^−1^ NP (Figure 3). CAT in *S. quadricauda* and *C. minutus* decreased 1.36- and 2.09-fold, respectively, at 1.5 mg L^−1^ of NP compared with the control. CAT in *A. acicularis* increased when NP was below 0.5 mg L^−1^, followed by a drop towards the control at 1.5 mg L^−1^. Peroxidase (POD) showed different patterns in comparison with SOD and CAT, so that the minimum activity was shown in the control group of *C. vulgaris*, followed by *C. minutus*, *S. bibraianum*, *S. quadricauda*, and *S. obliquus* (Figure 3).

In this study, the changes in antioxidants indicated that the microalgae were suffering from oxidative stress when exposed to NP (Figure 3). SOD in algae decreased when the NP concentration was high, indicating that NP significantly inhibited SOD production to eliminate H_2_O_2_. The increase of CAT and POD indicated that CAT and POD also contributed to the removal of H_2_O_2_. In comparison with NP-sensitive species, a recent study showed similarly that *C. vulgaris* is an NP-tolerant species, as exhibited by a higher and more rapid increase in CAT [35].

### 2.4. NP Accumulation and Degradation in Algae

**Figure 4:**
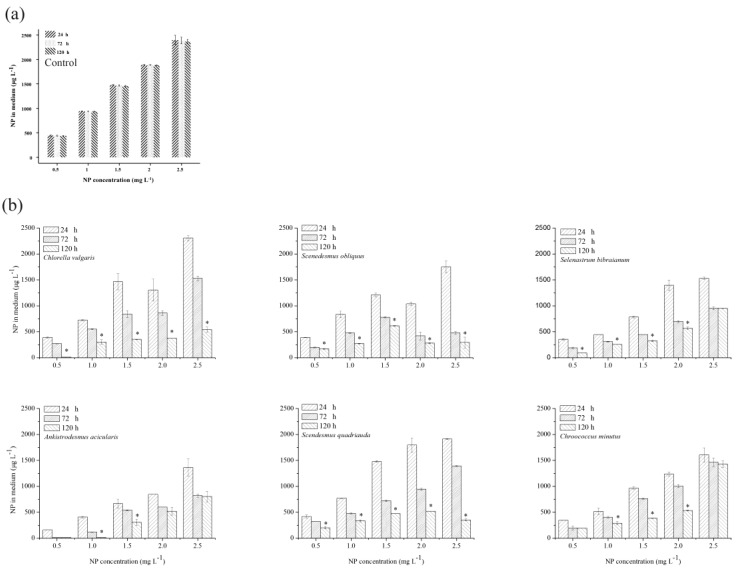
Residual NP in the medium: (**a**) control and (**b**) six algae. Algae were treated with NP at 0.5–2.5 mg L^−1^ culture for 120 h. Values are the mean ± standard deviation (SD) (*n* = 3). Asterisks indicate the significant differences compared to other NP treatments (*p* < 0.05).

**Figure 5:**
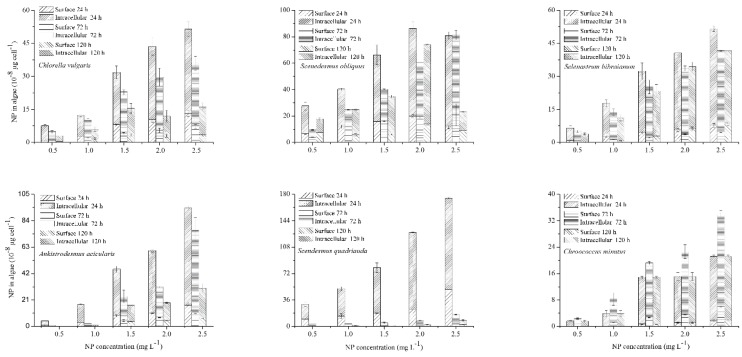
NP accumulation in microalgae. Algae were treated with NP at 0.5–2.5 mg L^−1^ culture for 120 h. Values are the mean ± standard deviation (SD) (*n* = 3).

It is known that NP is removed from water solutions in light by abiotic degradation [33]. The residual concentrations of NP in the medium of the control flasks did not show any significant changes during the 120 h experiments (Figure 4a), indicating that the abiotic loss was negligible. On the contrary, the NP level decreased rapidly when algae were available (Figure 4b). To fully evaluate the fate of NP in algae, target compounds were detected under different treatments. NP accumulation increased with the enhancement of the initial NP level (Figure 5). To confirm the accumulation of NP, the residual NP concentration in the medium was evaluated by a time-dependent study demonstrating a gradual decrease of NP over time (Figure 4b). In addition, the medium containing algae had a lower amount of NP compared with the control, suggesting that a certain proportion of NP had accumulated both on the surface and in the interior of the algae.

The quantity of NP removed from the medium was higher than the accumulated amount in algal cells due to the apparent biodegradation of NP. The maximum biodegradation percentages of *C. vulgaris*, *S. obliquus*, *S. bibraianum*, *A. acicularis*, *S. quadricauda*, and *C. minutus* in the present study were 89.5%, 52.5%, 63.1%, 95.6%, 84.6%, and 44.6%, respectively, indicating that the biodegradation of *C. vulgaris*, *A. acicularis*, and *S. quadricauda* was much faster than that of *Microcystis aeruginosa*, where more than 60% of NP degraded [11,40], and was also faster than other microalgae [40]. The six algae in this study varied in their biological degradation capability. *C. vulgaris*, *S. bibraianum*, and *A. acicularis* decreased with the increase of the initial NP level, and the biodegradation of NP reached the minimum at 2.5 mg L^−1^ (62.4%, 32.8%, and 34.9%, respectively). Therefore, *A. acicularis* was the most effective species for NP biodegradation when the NP concentration was below 1.0 mg L^−1^, indicating that NP biodegradation was associated with algal growth, a finding similar to that of a previous study by Yan et al. on *Chlorella pyrenoidosa* [41]. Further, in natural water bodies, surface water containing more than 10 μg/L NP is considered highly polluted, water containing 1–10 μg/L is polluted, and surface water containing <1 μg/L of NP has a low pollution level [42]. Even if severe NP pollution occurred in water bodies, the NP concentration was only up to 325 μg L^−1^ [43]. Obviously, the NP concentration of 1 mg L^−1^ could cover most of the polluted concentration of water bodies in nature. So, it would appear that *A. acicularis* could be applied in sewage treatment for its potential to effectively remove NP from water bodies in a suitable scope.

**Figure 6:**
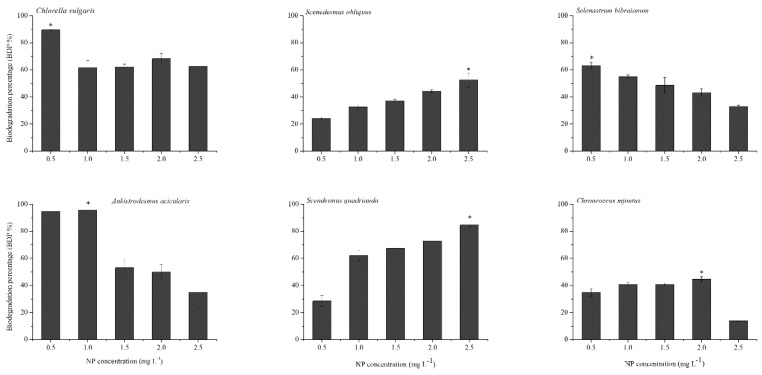
Biodegradation of NP by microalgae. Algae were treated with NP at 0.5–2.5 mg L^−1^ culture for 120 h. Asterisks indicate the significant differences compared to other NP treatments (*p* < 0.05).

The algae in this study grew well at 0.5-2.5 mg L^−1^ NP. The concentration of NP decreased significantly during the exponential growth phase (Figure 1 and Figure 4). In contrast, the biodegradation by *S. quadricauda* and *S. obliquus* increased with an increase in the initial NP concentration. *S. quadricauda* was the most effective species for NP biodegradation when the NP concentration was 2.5 mg L^−1^ (Figure 6). However, the growth was inhibited when NP was higher than 1.0 mg L^−1^. The metabolism of other phenolic compounds in microalgae displayed similar patterns in comparison with higher plants. Some freshwater microalgae can metabolize bisphenol-A (BPA) to BPA glycosides, which are released into the culture medium [44]. In *Tetraselmis marina*, the metabolism of *p*-chlorophenol (*p*-CP) includes glucosyl transfer followed by malonyl transfer [45]. NP biodegradation in bacteria has been broadly reported, but similar research on microalgae is scarce. Some by-products during biodegradation are disadvantageous to algae growth. For example, 4-n-nonylphenol, a by-product of 4-n-nonylphenol, was degraded by *Metarhizium sp.* [46] and inhibited growth of the green alga *Chlorella sorokiniana* at 0.30 mg L^−1^ [47]. In our study, some by-products during biodegradation blocked *S. quadricauda* growth.

## 3. Materials and Methods

### 3.1. Reagents

Nonylphenol was purchased from Sigma-Aldrich (St. Louis, MO, USA). Methyl alcohol and acetonitrile were chromatographically pure, from Shanghai Anpu Company (Shanghai, China). Other reagents such as those used in culture media were analytical grade, from Guangzhou Chemical Factory (Guangzhou, China).

### 3.2. Algal Culture and Treatment

Six freshwater microalgae obtained from NP-polluted water at Jinan University, Guangzhou, China were identified using “The freshwater algae of China” (Hu and Wei, 2006). *C. vulgaris* (JNU38), *S. obliquus* (JNU15), *S. bibraianum* (JNU28), *A. acicularis* (JNU14), *S. quadricauda* (JNU39), and *C. minutus* (JNU17) were isolated. The algae were kept individually in conical flasks (2 L) in 1 L of BG11 medium with constant shaking (100 rpm) at 25 ± 2 °C under cool white fluorescent lamps (80 μmol m^−2^ s^−1^) at a 12 h light:12 h dark regime. All containers and solutions prior to utilization were autoclaved for 15 min at 121 °C. BG11 medium included the basal culture and trace metal medium [48]. The basal culture medium contained 1.5 g L^−1^ NaNO_3_, 40 mg L^−1^ K_2_HPO_4_, 75 mg L^−1^ MgSO4·7H_2_O, 36 mg L^−1^ CaCl_2_·2H_2_O, 20 mg L^−1^ NaHCO_3_, 6 mg L^−1^ ferric ammonium citrate, and 6 mg L^−1^ citric acid. The trace metal solution contained 2.86 mg L^−1^ H_3_BO_3_, 1.81 mg L^−1^ MnCl_2_·4H_2_O, 222 mg L^−1^ ZnSO4·7H_2_O, 390 mg L^−1^ Na_2_MoO_4_·2H2O, 79 mg·L^−1^ CuSO_4_·5H_2_O, and 49.4 mg L^−1^ Co(NO_3_)_2_·6H_2_O.

### 3.3. Removal of Bacteria from Algal Cultures

After 100 mL algal cultures in mid-exponential phase were filtered through a 10 μm pore size membrane, they were suspended in 50 mL of sterile BG11 medium, followed by 10 min centrifugation at 1000× *g*. The cells were then washed three times and suspended in sterile medium (50 mL) containing 0.1 M EDTA and 0.005% Tween-80 for 1 h at 20 °C, after which 0.5 mg mL^−1^ lysozyme (LSZ) and 0.25% sodium dodecyl sulphate (SDS), which had been warmed for 10 min at 20 °C, were added in sequence. Thereafter, cells were centrifuged at 1000× *g* for 10 min, washed two times to eliminate SDS and LSZ, and resuspended in 50 mL of sterile medium. The antibiotics kanamycin (50 μg mL^−1^) and penicillin (100 μg mL^−1^) were added to the algal cultures, which were maintained under a 12h light:12h dark regime at 20 °C for 1 week. Bacterial presence was evaluated, after subculturing three times, by epifluorescence microscopy using 4′,6-diamidino-2-phenylindolestain (DAPI) stain, ensuring a sterile condition [49,50].

### 3.4. Nonylphenol Treatments

The stock solution of NP was prepared in methanol at a concentration of 1000 mg L^−1^. Working solutions were set up at concentrations of 0, 0.5, 1.0, 1.5, 2.0, 2.5, and 3.0 mg L^−1^. Every group was in triplicate. The control group was treated with an equivalent amount of methanol (0.1%). The final concentration of methanol was controlled at 0.40% (*v*/*v*) for all experimental media in order to eliminate the effect of methanol on algal cells [51].

All solutions and experimental containers were autoclaved at 121 °C for 15 min. The microalgal cultures in the middle of the log phase of growth were decanted into 100 mL flasks containing 40 mL of medium at 25 ± 2 °C and illuminated with fluorescent lights (90 mol m^−2^ s^−1^ photon flux intensity) under a 12:12 h light:dark photoperiod. The algae were transferred to flasks (150 mL) containing 100 mL of BG11 medium, with the initial algal densities of 1–3 × 10^5^ cell m L^−1^. The experiments lasted for 5 days (120 h) with intermittent shaking.

### 3.5. Determination of Algal Growth and Inhibitory Effect Concentration

Cell density was indirectly measured by chlorophyll a, and regression equations between cell density and chlorophyll content of the six algae were prepared. A light microscope (Olympus, Japan) was used. Chlorophyll in vivo was evaluated by a TD-700 fluorometer (Turner Design, Fresno, CA, USA) after calibration with a chlorophyll standard. Before measuring, tubes with 10 mL cultures were kept for 20 min in complete darkness at room temperature (RT) followed by constant shaking (100 rpm) three times. Chlorophyll concentration was measured with 420 nm excitation and 680 nm emission spectrum. The content of chlorophyll a was measured at 0, 24, 48, 72, and 96 h.

EC_50_ was defined as the NP concentrations until the chlorophyll content diminished by half. EC_50_ at 96 h was calculated by a probability unit and concentration logarithm [52]. The algal growth rate (μ) was calculated according to the following equation:

µ (d − 1) = (Ln Nt − Ln N0)/(t − t0)(1)

where *N_0_* and *N_t_* are the cell density at the beginning (*t_0_*) and the end (*t*) of the selected time interval, respectively.

The percentages (*I_r_*) of algal chlorophyll reduction were calculated according to Equation (2), and the *Ir* (%) was then used to calculate the EC_50_ value based on chlorophyll a:

*I_r_* (%) = 100 × (*y*_c_ − *y*_s_)/*y*_s_ (2)

where *y_c_* and *y*_s_ are the algal growth rates or chlorophyll a contents in the control medium and test medium, respectively.

According to the probability unit and concentration logarithm, linear regressions were set up and used to obtain EC_50_ values. Origin software version 8.0 (Microcal Software Inc., USA) was applied.

### 3.6. Measurement of Fluorescence Transient

A plant efficiency analyzer (PEA; Hansatech Instruments Ltd., UK) was applied for chlorophyll fluorescence transient analysis at 96 h. The algae were placed in complete dark for 20 min at RT. Aqueous-phase attachment of the PEA was applied for measurement. A red light of 3500 µmol photons m^−2^ s^−1^ provided by an array of six high-intensity light-emitting diodes was applied to generate transients measured on a time scale from 10 ms to 1 s.

The fluorescence intensity at 50 ms was defined as the initial fluorescence (*F_0_*), the peak fluorescence was determined as *F_m_*, and the difference between *F_m_* and *F_0_* (*F_m_* − *F_0_*) was defined as *F_v_*. *F_v_*/*F_m_* was calculated by (*F_m_* − *F_0_*)/*F_m_* [53].

### 3.7. Assay of Antioxidant Enzyme Activity

After 96 h, algae were harvested following 10 min of centrifugation at 3000× *g*. The algae were then added to 2 mL of ice-cold extraction buffer including Tris–HCl (50 mM, pH 7.8), EDTA (1 mM), ascorbate (1 mM), as well as polyvinylpyrrolidone (1.5%, *w*/*w*). Then, the cocktails were centrifuged at 15,000× *g* for 20 min at 4 °C. The supernatants were used to estimate the activities of antioxidant enzymes. SOD (EC1.1.5.1.1) was determined by photochemical inhibition of nitro blue tetrazolium (NBT) [12]. CAT (EC 1.11.1.6) was spectrophotometrically estimated by the ammonium molybdate method [12]. POD activity was evaluated at 420 nm [54].

### 3.8. Quantification of NP in Culture Medium and Algae

The concentrations of NP in the medium were measured at 24, 72, and 120 h, and algae only at 120 h. Liquid–liquid microextraction (DLLME) [50] was applied during measurement. In brief, a sample (5 mL) was mixed with 0.2 mL of chlorobenzene and acetone (1:2), with a milky cloudy mixture generated after gentle shaking, and then subsequently centrifuged at 4500× *g* (15 min, 4 °C). A microsyringe (50 μL, zero dead volume, cone-tip needle) was used to withdraw the dispersed fine particles of the extraction phase that settled at the bottom of the conical test tube, repeated three times. The sediment fractions were combined for further analysis by high-performance liquid chromatography (HPLC) (Agilent, Santa Clara, CA, USA) [20]. All the extraction was performed at room temperature (23 ± 2 °C).

Before DLLME, cells were separated from the 5 mL cultures withdrawn from the flasks through centrifugation at 4500× *g* for 15 min at 4 °C. NP concentration in the culture medium could be measured by analyzing the NP distribution in the supernatant using DLLME and HPLC. The cell pellets were washed with 5 mL of 10% methanol with shaking for approximately 60 s, and the wash water was separated and used to analyze the NP adsorbed on the surface [40]. The cell pellet was mixed with anhydrous Na_2_SO_4_ and dichloromethane-methanol (1:2 *v*/*v*, 3 mL) by sonication for 20 min and centrifuged for 5 min at 3500× *g*. The extraction was done three times and the solvent fractions were combined for the analysis of NP absorbed by cells [55].

### 3.9. Determination of Biodegradation Percentage of NP

The biodegradation percentage (BDP) of NP was calculated as

BDP (%) = 100 × (C_i_ − C_r_ − C_a_ − C_d_ × W_a_ − C_c_ × W_a_) / C_i_ (3)

where C_i_ is the NP initial concentration in the solution (mg L^−1^), C_r_ is the NP residual concentration in the solution (mg L^−1^), C_a_ is the NP in abiotic elimination (mg L^−1^), C_d_ is the concentration (mg g^−1^) dry weight of NP adsorbed on the cell wall, C_c_ is the concentration (mg g^−1^) dry weight of NP accumulated in algal cells, and W_a_ is the dry mass of algae (g L^−1^) [49].

### 3.10. Statistical Analysis

All data in this research are presented as mean ± standard deviation (SD) (*n* = 3). One-way analysis of variance (ANOVA) was applied to identify the differences among treatments, followed by the least significant difference (LSD) test if the ANOVA result was significant (*p* < 0.05). The statistical analyses were performed with SPSS 12.0. The linear correlation was performed with Origin 8.0 using the least-squares fitting method.

## 4. Conclusions

Both the concentration and exposure time of NP were found to affect the responses of microalgae. High levels of NP (≥1.5 mg L^−1^) were highly toxic to all microalgae in which various antioxidant mechanisms were involved. *C. vulgaris* and *S. obliquus,* which are NP-tolerant species, responded rapidly to antioxidation compared with NP-sensitive species, especially when the NP concentration was high. The result that NP showed strong acute toxicity to *C. minutus* suggests that it could be a promising tool for the study of NP toxicity. All six microalgae species biodegraded NP at a low concentration but in a species-specific manner. Our results indicate that *A. acicularis* degraded NP when the concentration was below 1.0 mg L^−1^, while *S. quadricauda* more actively biodegraded NP when the concentration was above 1.5 mg L^−1^. However, considering NP pollution in natural water and algal growth, *A. acicularis* is more suitable to be applied in sewage treatment.

## Figures and Tables

**Table 1:** Acute toxicity of NP (mg L^−1^) on six microalgae species at 96 h culture time.

Microalgal Species	Regression Equation	*R* ^2^	EC_50_ (mg L^−1^)
*Chlorella vulgaris*	y = 0.875x + 0.1257	0.975	1.534
*Scenedesmus* *obliquus*	y = 1.716x + 0.2182	0.904	1.179
*Selenastrum* *bibraianum*	y = 1.6141x + 0.237	0.961	1.177
*Ankistrodesmus* *acicularis*	y = 1.3231x + 0.374	0.911	1.100
*Scenedesmus* *quadricauda*	y = 1.3366x + 0.3974	0.961	1.080
*Chroococcus* *minutus*	y = 1.1814x + 0.4941	0.970	1.005

The concentrations of NP were in the ranges of 0.5–3.0 mg L^−1^. EC_50_: the median inhibitory effect concentration (mg L^−1^). *R*^2^: correlation coefficient. *p*-value significance of linear regression with 95% confidence limits in ANOVA.

**Table 2:** The 96 h EC_50_ of NP on different microalgae.

Microalgal Species	EC_50_ (mg L^−1^)	Reference
*Microcystis* *aeruginosa*	0.67–2.96	[27]
*Dunaliella* *salina*	1.47	[28]
*Scenedesmus* *obliquus*	1.0	[29]
*Scenedesmus* *subspicatus*	0.87–0.98	[30]
*Phaeocystis* *globosa*	0.42	[31]
*Skeletonema* *costatum*	0.13	[32]
*Chaetoceros* *curvisetus*	0.22	[32]
*Cyclotella* *caspia*	0.18	[33]
